# Suicidal Expression among School-Attending Adolescents in a Middle-Income Sub-Saharan Country 

**DOI:** 10.3390/ijerph9114122

**Published:** 2012-11-13

**Authors:** Michael L. Wilson, Andrea C. Dunlavy, Bharathi Viswanathan, Pascal Bovet

**Affiliations:** 1 Centre for Injury Prevention and Community Safety (CIPCS), PeerCorps Trust Fund, P.O. Box 22499 Dar es Salaam, Tanzania; Email: andrea.dunlavy@peercorpstrust.org; 2 Ministry of Health, Victoria, Republic of Seychelles; Email: barathi.viswanathan@health.gov.sc; pascal.bovet@chuv.ch; 3 Institute of Social and Preventive Medicine (IUMSP), Lausanne University Hospital (CHUV) Lausanne 1010, Switzerland

**Keywords:** suicidal expression, adolescent, school health, sub-Saharan Africa

## Abstract

We investigated correlates for suicidal expression among adolescents in the Seychelles. Data on 1,432 students (52% females) were derived from the Global School-based Health Survey. Participants were divided into three groups: those with no suicidal behavior (N = 1,199); those with suicide ideation/SI (N = 89); and those reporting SI with a plan to carry out a suicide attempt/SISP (N = 139), each within a 12-month recall period. Using multinomial logistic regression, we examined the strength of associations with social, behavioral and economic indicators while adjusting for covariates. Sixteen percent of school-attending adolescents reported a suicidal expression (10% with a plan/6.2% without). Those reporting SI were younger (relative risk ratio RRR = 0.81; CI = 0.68–0.96), indicated signs of depression (RRR = 1.69; CI = 1.05–2.72) and loneliness (RRR=3.36; CI =1.93–5.84). Tobacco use (RRR = 2.34; CI = 1.32–4.12) and not having close friends (RRR = 3.32; CI = 1.54–7.15) were significantly associated with SI. Those with SISP were more likely to be female (RRR = 0.47; 0.30–0.74), anxious (RRR = 3.04; CI = 1.89–4.88) and lonely (RRR = 1.74; CI = 1.07–2.84). Having no close friends (RRR = 2.98; 1.56–5.69) and using tobacco (RRR = 2.41; 1.48–3.91) were also strongly associated. Having parents who were understanding was protective (RRR = 0.50; CI = 0.31–0.82). Our results suggest that school health promotion programs may benefit from targeting multiple factors associated with suicidal expression. More research, particularly multilevel designs are needed to identify peer and family influences which may modify associations with suicidality.

## 1. Introduction

Suicide ranks as the third leading cause of mortality among adolescents worldwide [1], and according to recent research, suicidal expression may be on the rise [2]. While few cross-nationally representative studies of suicide exist, one study among 34 nations documented that suicide was responsible for the deaths of more than 15,000 adolescents during a one-year period [3]. 

Suicidal expression has long been associated with several psychosocial indicators, many of which are intensified during adolescence [4]. Some of these include poor mental health, poverty, being bullied, substance use and having poor relationships with parents [5,6,7,8]. While most suicide attempters are female, males are overrepresented in suicide completions [9]. These indicators however, are largely representative of adolescents in high-income country (HIC) settings where surveillance data have generally greater accuracy. Among low- and middle income country (LMIC) settings however, the picture remains less clear, particularly in countries of the African region [10]. For example some countries report data which are not disaggregated by age, making estimations of suicide among adolescents difficult. In young people, death by suicide is often obscured by other mortality diagnoses or documented as being accidental. Cultural and religious barriers also make it difficult to classify a death as a suicide. Even the ascertainment of suicide by health care professionals is not uniform in all settings [9,11]. 

Adolescents who attempt a suicide and fail may injure themselves seriously enough to require medical attention. The trauma which follows an unsuccessful attempt may be physically disfiguring, or result in long term disability. The mental health consequences of attempts are equally significant, and there are fewer rehabilitative resources available in LMIC settings. Subsequent to an attempt, the associated stigma or physical scarring may affect school attendance. Those who fail to return and complete school are less likely to find employment as adults. Parents, often faced with few treatment options, may be forced to reduce meaningful economic activity to reintegrate their adolescent into the family and community [11]. Among poor households, this means being driven deeper into poverty [12]. Despite this, research on correlates for suicidal expression among adolescents in LMICs remains a neglected public health priority. The aim of this study was to examine the social and demographic factors associated with two forms of self-reported suicidal expression among adolescents in a middle-income sub-Saharan country.

## 2. Methods

### 2.1. Setting

This study is based on data collected in the Republic of Seychelles. The Seychelles constitutes a group of islands located approximately 1,800 km east of Kenya. With a gross domestic product of USD $8,000 in 2007, the Seychelles is considered an upper middle-income country [13].

### 2.2. Sample

The data for this study were drawn from the Seychelles contribution to the Global School-based Student Health Survey (GSHS). The GSHS was developed by the World Health Organization in collaboration with the US Centers for Disease Control. It collects relevant information for the discernment of behavioral and health risks among adolescents of school age in 43 countries. In the Seychelles, 1,432 secondary school students (52% females) aged 11–17 years participated in the survey which had a response rate of 82%. We excluded five participants that did not have complete information resulting in a final sample of 1,427 participants. Detailed information on data collection methods, the questionnaire and procedures are published elsewhere [14]. At the time of data collection, the research committee of the Ministry of Health and the Ministry of Education approved the study including the questionnaire. Informed consent by parents was not necessary.

### 2.3. Measurements

Participants were divided into three groups: those not reporting suicidal expression (N = 1,199); those reporting suicide ideation but not suicide planning (SI group; N = 89), and those reporting both SI and planning (SISP group; N = 139) each with a 12-month period of recall. We derived our dependent variables from the responses to two questions in the GSHS: *“During the past 12 months, did you ever seriously consider attempting suicide?”* and *“During the past 12 months, did you make a plan about how you would attempt suicide?”* The response options were *“yes/no”*. Those who responded *“yes”* to SI and *“no”* to planning were considered part of the ideation only group. Those who responded *“yes”* to planning regardless of whether they had prior SI were considered part of the SISP group.

Our analyses targeted contextually relevant demographic, psychosocial and family factors which have been found to be associated with suicidal expression in the peer-reviewed literature. These factors included: food deprivation, anxiety, loneliness, signs of depression, social support (friendships), truancy, bullying, parent involvement, tobacco use, substance use and alcohol misuse [5,6,8,15,16]. Based on these, the following survey questions served as the basis for the selection of independent variables.

To examine associations with food deprivation, anxiety and loneliness we used: “*During the past 30 days, how often did you go hungry because there was not enough food in your home?*”, “*During the past 12 months, how often have you been so worried about something that you could not sleep at night?*” and “*Duringthe past 12 months, how often have you felt lonely?*”. The response options to each of these questions were “*never*”, “*rarely*”, “*sometimes*”, “*most of the time*”, or “*always*”. These were dichotomized into “*yes*” corresponding to “*most of the time/always*” and “*no*” corresponding to “*never/rarely/sometimes*”. To examine associations with signs of depression we used “*During the past 12 months, did you ever feel so sad or hopeless almost every day for two weeks or more in a row that you stopped doing your usual activities?*”. The responses were “*yes/no*”. For social support we used “*How many close friends do you have?*” Response options were “*0*”, “*1*”, “*2*”, and “*3 or more friends*”.

We examined associations with truancy by using: “*During the past 30 days, on how many days did you miss classes or school without permission?*”. Response items were “*0*”, “*1–2*”, “*3–5*”, “*6–9*” and “*10 or more days*”. Students were considered truant if they missed more than 3 days of school within the reference period using a prior threshold by Wilson *et al.*[17]. To examine associations with experiences of being bullied, the following was used: “*During the past 30 days, on how many days were you bullied?*” Response items were “*0*”, “*1–2*”, “*3–5*”, “*6–9*”, “*10–19*”, “*20–29*” and “*all 30 days*”. One category for bullying in the last 30 days was created using three or more days. For parental involvement in school, parent-child relationship quality and whether parents were knowledgeable about their child's leisure time activities we used: “*During the past 30 days how often did your parents or guardians check to see if your homework was done?*”, “*During the past 30 days, how often did your parents or guardians understand your problems and worries?*” and “*During the past 30 days, how often did your parents or guardians really know what you were doing with your free time?*”. The response options to each of these were “*never*”, “*rarely*”, “*sometimes*”, “*most of the time*”, and “*always*”. These were dichotomized into “*most of the time/always*”, which was re-coded as a “*yes*” response, for comparison with “*never/rarely/sometimes*”, or a “*no*” response. 

Associations with tobacco use were examined using: *“During the last 30 days, on how many days did you smoke cigarettes?”*. The responses options were: *“0”, “1*–*2”, “3*–*5”, “6*–*9”, “10*–*19”*, and *“all 30 days”*. One category for any cigarette use within the past 30 days was created using one or more days. Associations with substance use were examined using: *“During your life, how many times have you used drugs such as marijuana, cannabis or hashish, steam, stuff, joint, lapay or tyalas (“lapay” and “tyalas” are Creole names for marijuana)?”* The response items were *“0”, “1–2”, “3*–*9”* and *“10 or more times”*. One category for any lifetime substance use was created using one or more days. Finally, we examined associations with lifetime alcohol misuse by using: *“During your life, how many times have you ever had a hang-over, felt sick, got into trouble with your family or friends, missed school, or got into fights, as a result of drinking alcohol?”*. Responses were *“0”, “1*–*2”, “3*–*9”* and *“10 or more times”*. One category for lifetime alcohol misuse was created using one or more days.

### 2.4. Statistical Analysis

We first examined the distribution of selected variables within each of the three suicide expression categories. Significant differences between each category and independent variables were explored using Pearson’s chi-square for categorical variables and ANOVA for continuous variables (age). We then used two multinomial logistic regression (MLR) models to examine independent variable associations with those without suicidal expression, those with SI and those reporting SISP while adjusting for covariates. Compared with binary logistic regression, MLR is an extension which allows for the prediction of the probabilities of more than two outcomes of a categorically distributed dependent variable. Given that our dependent variable had three categories, MLR was more suitable than a standard binary logistic regression. In the first MLR model, we included all variables which were statistically significant in the bivariate analyses at *p* < 0.05. In the second model we adjusted for age and sex. The measures of association were reported as relative risk ratios (RRR) along with their with 95% confidence intervals (CI). All analyses were conducted using Stata for Linux, version 12 (StataCorp, College Station, TX, USA, 2011).

## 3. Results

Within the recall period, 16% of adolescents reported suicidal expression, 10% of whom reported a plan and 6.2% of whom reported ideation alone. Sixty-eight percent of those who reported having made a plan to carry out a suicide attempt were female. The mean age of the sample was 14.0 (SD 1.5). Thirty-percent of respondents reported signs of depression, with 14% indicating loneliness and 12% percent anxiety. Thirty-six percent of respondents reported alcohol misuse, 19% reported substance use at least once during their lifetimes and 23% had used tobacco. Slightly more than 18% reported being deprived of food within the 30 days prior to the survey.

Table 1 shows the crude distribution of selected factors according to suicide expression category. We found significant differences across all categories of psychological health and the broad category of substance use. Each were associated with higher levels of SI and SISP. Males were overrepresented among those with suicidal ideation (*p* < 0.001) as were those who reported being bullied three or more days during the recall period (*p* < 0.001). Adolescents who had parents who were understanding of their worries or concerns, were significantly less likely to report SI or SISP (*p* < 0.001). We found what appeared to be a linear association between the number of close friends reported and the rate of suicidal ideation and planning (*p* < 0.001). No significant differences were found with respect to age, truancy or whether parents had spent time assisting their adolescents with homework tasks.

**Table 1:** Distribution of selected factors according to categories of suicide expression among school-attending adolescents in the Seychelles.

Variable	No suicide expression (n = 1,199)	Suicide ideation (n = 89)	Suicide planning (n = 139)	*P*-value
Age (SD)	14.0 (1.5)	13.6 (1.5)	14.1 (1.4)	ns
Sex (male)	48.5	57.3	32.4	<0.001
Food deprivation	17.0	25.8	23.7	0.024
Anxiety	9.3	18.0	34.5	<0.001
Signs of depression	25.9	43.8	59.0	<0.001
Loneliness	10.2	32.6	30.2	<0.001
Bullied	34.5	53.9	50.4	<0.001
Truancy	18.7	24.7	22.3	ns
Parent helps with homework	44.7	47.2	35.3	ns
Parent knowledgeable about free time	45.1	47.2	33.1	0.021
Parents were understanding	43.0	37.1	23.7	<0.001
Tobacco use	20.0	37.1	36.0	<0.001
Substance use	18.0	25.8	23.7	0.040
Alcohol misuse	21.7	34.8	33.1	<0.001
Close friends				
0 friend	4.1	12.6	14.5	<0.001
1 friend	14.7	16.1	15.9	-
2 friends	17.1	18.4	20.3	-
≥3 friends	64.1	52.9	49.3	-

All variables are expressed as proportions (in %) except for age (mean and standard deviation).

After adjusting for all associated covariates (Table 2), and compared to those without suicide expression, those reporting SI were younger (RRR = 0.81; CI = 0.68–0.96), more likely to report signs of depression (RRR = 1.69; CI = 1.05–2.72) and feel lonely (RRR = 3.36; CI = 1.93–5.84). We also found significant associations with tobacco use (RRR = 2.34; CI = 1.32–4.12) and not having close friends (RRR = 3.32; CI = 1.54–7.15). Those with SISP were more likely to be female (RRR = 0.47; CI = 0.30–0.74), anxious (RRR = 3.04; CI = 1.89–4.88) and lonely (RRR = 1.74; CI = 1.07–2.84). Having no friends (RRR = 2.98; 1.56–5.69) and using tobacco (RRR = 2.41; 1.48–3.91) were also strongly associated with SISP. Having parents who were understanding was protective (RRR = 0.50; CI = 0.31–0.82). Initial associations with food deprivation, bullying, substance use and alcohol misuse disappeared once other covariates were included in the model. 

In Table 3, we show the results for the analysis adjusted for age and sex. Those who reported SI only were more anxious (RRR = 1.98; CI = 1.11–3.54), depressed (RRR = 2.26; CI = 1.46–3.53) and lonely (RRR = 4.33; CI = 7.04). Being bullied (RRR = 2.00; CI = 1.29–3.11) was significantly associated as well as tobacco (RRR = 2.30; CI = 1.44–3.67) and alcohol misuse (RRR = 1.85; CI = 1.16–2.94). Not having friends (RRR = 3.88; CI = 1.88–8.02) was also significantly associated with SI. For those reporting SISP, only having one (RRR = 1.33; CI = 0.80–2.22) or two (RRR = 1.44; CI = 0.90–2.30) friends were non-significant. 

**Table 2:** Multivariate analysis of suicide ideation and planning among school-attending adolescents in the Seychelles.

Variable	Suicidal ideation RRR (CI)	*P*-value	Suicidal planning RRR (CI)	*P*-value
Age	0.81 (0.68–0.96)	0.014	1.05 (0.92–1.21)	ns
Sex (male)	1.43 (0.87–2.34)	ns	0.47 (0.30–0.74)	0.001
Food deprivation	1.21 (0.70–2.08)	ns	1.12 (0.70–1.80)	ns
Anxiety	1.05 (0.54–2.02)	ns	3.04 (1.89–4.88)	<0.001
Signs of depression	1.69 (1.05–2.72)	0.032	2.68 (1.80–4.00)	<0.001
Loneliness	3.36 (1.93–5.84)	<0.001	1.74 (1.07–2.84)	0.026
Bullied	1.33 (0.82–2.14)	ns	1.41 (0.94–2.14)	ns
Parent knowledgeable about free time	1.33 (0.80–2.20)	ns	0.92 (0.59–1.44)	ns
Parents were understanding	0.71 (0.41–1.21)	ns	0.50 (0.31–0.82)	0.006
Tobacco use	2.34 (1.32–4.12)	0.003	2.41 (1.48–3.91)	<0.001
Substance use	0.83 (0.42–1.62)	ns	0.97 (0.55–1.69)	ns
Alcohol misuse	1.23 (0.74–2.22)	ns	1.28 (0.81–2.03)	ns
Close friends				
0 friend	3.32 (1.54–7.15)	0.002	2.98 (1.56–5.69)	0.001
1 friend	1.21 (0.63–2.33)	ns	1.09 (0.63–1.91)	ns
2 friends	1.26 (0.67–2.34)	ns	1.09 (0.65–1.83)	ns
≥3 friends (reference)	-	-	-	-

RRR = Relative Risk Ratio.

CI = 95% Confidence Interval.

All estimates are adjusted for all variables listed in the table.

**Table 3:** Multivariate analysis of suicide ideation and planning among school-attending adolescents in the Seychelles.

Variable	Suicidal ideation RRR (CI)	*P*-value	Suicidal planning RRR (CI)	*P*-value
Food deprivation	1.60 (0.97–2.64)	ns	1.55 (1.01–2.36)	0.043
Anxiety	1.98 (1.11–3.54)	0.020	5.23 (3.48–7.85)	<0.001
Signs of depression	2.26 (1.46–3.53)	<0.001	3.95 (2.75–5.69)	<0.001
Loneliness	4.33 (2.66–7.04)	<0.00	3.65 (2.42–5.50)	<0.001
Bullied	2.00 (1.29–3.11)	<0.002	2.21 (1.53–3.17)	<0.001
Parent knowledgeable about free time	0.75 (0.48–1.18)	ns	0.43 (0.29–0.65)	<0.001
Parents were understanding	0.75 (0.48–1.18)	ns	0.43 (0.29–0.64)	<0.001
Tobacco use	2.30 (1.44–3.67)	<0.001	2.76 (1.87–4.08)	<0.001
Substance use	1.51 (0.90–2.52)	ns	1.85 (1.19–2.86)	0.006
Alcohol misuse	1.85 (1.16–2.94)	0.010	2.03 (1.38–3.00)	<0.001
Close friends				
0 friend	3.88 (1.88–8.02)	<0.001	4.67 (2.60–8.37)	<0.001
1 friend	1.35 (0.72–2.52)	ns	1.33 (0.80–2.22)	ns
2 friends	1.38 (0.76–2.51)	ns	1.44 (0.90–2.30)	ns
≥3 friends (reference)	-	-	-	-

RRR = Relative Risk Ratio.

CI = 95% Confidence Interval.

All estimates are adjusted for age and sex.

## 4. Discussion

We found that suicide expression here (16%) was lower than reported rates elsewhere in the SSA region (20–36%) using a comparable period of recall [10,18,19,20], but similar to those in HIC settings [21]. Higher average living standards and greater stability in the Seychelles, as compared with countries in the region, may partially explain these differences [22]. A favorable social context may potentially mitigate suicidal expression among adolescents, including social cohesion linked to the Creole culture, comprehensive education and health care services which are available free of cost, high community involvement due to the small size of the country, and low unemployment.

Suicidality is reported in the literature as being a non-specific marker for a broad range of psychosocial distresses such as political unrest and poor life quality [23]. These have been previously shown to negatively impact mental health at the individual level, potentially to the extent of self-harm [24,25]. While SI in the present sample was less prevalent among older adolescents, no significant association between age and SISP was found when other covariates were added to the model. This is in contrast to previous research which suggests that suicidality among those at risk increases after age 14 years. This may be due to increasing social pressures and expectations about pending adulthood [26]. In the Seychelles however, several government sponsored efforts exist to allow, for example, to pursue post secondary education and to find employment for school leavers, which can ease the transition between adolescence and adulthood.

Females were more likely to have reported planning a suicide attempt. This was consistent with findings elsewhere in the region [18,19] and internationally [27,28]. Other gender sensitive research on suicide suggests that while females may be more likely to think about suicide, males were more likely to successfully complete a suicide attempt [29]. Signs of depression and loneliness were both associated with SI and SISP, a finding which was congruent with other research highlighting mental ill-health as one important factor in suicidality [30]. 

Unlike prior research which informed that food deprivation was associated with higher rates of SI [5], we were not able to replicate this finding in the present sample. One hypothesis might be that although some participants reported not having enough food in the home, they may have had access to food while in school. Additionally, in the tropical climate of the Seychelles, fruits and vegetables are widely accessible throughout the year. It is also plausible that other factors such as family or sibling support offer protective mechanisms which confound associations between suicidality and food deprivation [31].

Consistent with prior research, tobacco use was elevated among adolescents with suicidal ideation [32,33]. We found that tobacco was use significantly associated with higher rates of self-reported SI and SISP. This may be related to evidence which supports tobacco use being used as a coping mechanism for negative life events, anger and stress [34], or other stressful situations such as physical transformation, sexuality, independence and social pressures [4].

Our finding that not having close friends was associated with an increase in suicidal expression, was congruent with the literature [30,35]. Adolescence is a period during which the formation of peer groups and friendships is an important aspect of their psychosocial development. Not having friends or being excluded from desirable peer groups, can have negative consequences for individual well-being and mental health [36]. Parent influence on the social behavior of children decreases during adolescence, with increased influence from peers [37]. However, our results demonstrate that parents may too play an important supportive role in the socialization process. Having understanding parents in our sample was associated with lower rates of SISP. This potentially suggests that adolescents who have open communication with their parents may be more likely to address their concerns.

After adjusting for covariates, we found no association between suicidal expression and parent knowledge about their child’s free time activities. Other research presented mixed results upon examining the link between parent supervision and adolescent suicidal expression. For example, among 2,598 pre/early adolescents in the United States, it was found that parent supervision played a significant role in reducing suicidal expression, but mainly among girls [38]. Other research has suggested that low parental monitoring, was independently associated with increased suicide expression [39]. Another longitudinal study provided even more inconclusive information - that parental vigilance was associated with increased rates of suicidality [40]. 

We found that reported rates of suicidal expression did not increase among those who reported being bullied, a finding not consistent with the literature [41]. Prior research from the Seychelles using similar measures and a similar period of recall, found that mental well-being declined in the presence of bullying [17]. However, this decline in mental health, may not have been sufficiently severe as to demonstrate higher levels of suicidal expression. It is also plausible that bullying rates among those who were victimized were underreported, thus obscuring the association with suicidal expression.

Despite research highlighting increased substance and alcohol use among those with suicidal expression [42,43], we were unable to replicate previous research findings using the current sample. One hypothesis concerning alcohol, may be the fact that other research considers alcohol use and not alcohol misuse as a correlate as was done in the present study. Thus there still exists the discrete possibility that alcohol use may be correlated with suicidal expression in the current sample, but misuse was not. The lack of an association with substance use may be explained by limitations in the data. Roughly 14% of all respondents reported some form of substance use during their lifetimes. It is likely that most of the substance use was due to experimentation, rather than as a coping mechanism in the presence of mental ill health. Furthermore, it is difficult to assess alcohol intake by use of a questionnaire, and imprecision in assessment of alcohol intake tends to drive an association to the null [44].

## 5. Limitations

This study provides valuable insight into a rarely explored public health phenomenon among adolescents in LMICs, and specifically in a sub-Saharan middle-income setting. To our knowledge this is the only study to have examined suicidal expression among adolescents in the Seychelles. Despite this, the results must be viewed in light of several limitations. First, the study remains silent on suicidal expression among adolescents who were absent on the day of the survey or who do not attend school. A previous survey in among school-attending adolescents in the Seychelles showed markedly higher unhealthy behaviors among students absent from school [45]. Secondly, although this survey was designed to be administered cross-culturally, there may have existed ambiguity in how questions were interpreted by respondents. For example, questions concerning mental health such as signs of depression, anxiety, or loneliness may manifest themselves differently cross culturally [46], and as such the reported symptoms may not be accurately captured when using Western measurement scales. Suicidal expression by peers and family has been documented as an important correlate for suicidal expression among adolescents. Thus the inclusion of information on family and peers in the survey would have strengthened the study [40]. Further research with culturally competent, standardized, multiple item measurements for signs of depression, anxiety, loneliness, as well as parent-child relationship quality are recommended. 

Finally, cultural taboos against suicidal expression may also have skewed our findings in the form of an underreporting bias. The 12-month recall period for suicidal expression may be subject to recall or social desirability biases. The cross-sectional design of the study did not allow for the determination of causal relationships between suicidal expression and associated factors. Finally, all items in the questionnaire were self-reported. Because the body of research on suicide expression in sub-Saharan settings is limited at present, the results should be limited to self-reported SI and SISP in the school-based adolescent population. 

## 6. Conclusions

Our results suggest that school health programs in the Seychelles may benefit from simultaneously targeting multiple factors associated with suicidal expression. Efforts should take into account the range of psychosocial characteristics of school-attending adolescents who report suicidal expression such as mental well-being, interaction with peers and substance use behaviors. Further research would benefit by making use of culturally appropriate and validated questionnaires to allow for enhanced reliability in examining factors such as parent-child relationship and mental health. Multilevel research designs are also suggested to identify peer and family influences which may modify associations with suicidality. The findings presented here may also be used to augment programs which emphasize the importance of healthy peer relationships, and help in the creation of environments in which healthy peer relationships can form. 
